# Experimental Study of the Propagation Process of Dissection Using an Aortic Silicone Phantom

**DOI:** 10.3390/jfb13040290

**Published:** 2022-12-09

**Authors:** Qing-Zhuo Chi, Yang-Yang Ge, Zhen Cao, Li-Li Long, Li-Zhong Mu, Ying He, Yong Luan

**Affiliations:** 1 Key Laboratory of Ocean Energy Utilization and Energy Conservation of Ministry of Education, Dalian University of Technology, Dalian 116024, China; 2 Department of Anesthesiology, The First Affiliated Hospital of Dalian Medical University, Dalian 116011, China

**Keywords:** 3D printing, silicone phantom, CT scanning, aortic dissection, interlayer adhesion damage

## Abstract

Background: The mortality of acute aortic dissection (AD) can reach 65~70%. However, it is challenging to follow the progress of AD formation. The purpose of this work was to observe the process of dissection development using a novel tear-embedded silicone phantom. Methods: Silicone phantoms were fabricated by embedding a torn area and primary tear feature on the inner layer. CT scanning and laser lightening were conducted to observe the variations in thickness and volume of the true lumen (TL) and false lumen (FL) during development. Results: The model with a larger interlayer adhesion damage required a lower pressure to trigger the development of dissection. At the initiation stage of dissection, the volume of TL increased by 25.5%, accompanied by a 19.5% enlargement of tear size. The force analysis based on the change of tear size verified the deduction of the process of interlaminar separation from the earlier studies. Conclusions: The primary tear and the weakening adhesion of the vessel layers are key factors in AD development, suggesting that some forms of primary damage to the arterial wall, in particular, the lumen morphology of vessels with straight inner lumen, should be considered as early risk predictors of AD.

## 1. Introduction

Acute aortic syndrome is a group of interrelated life-threatening diseases that includes aortic dissection (AD), intramural hematoma, and penetrating atherosclerotic ulcer. The disease of the aortic wall develops rapidly and can present as chest tightness and chest or back pain. The incidence of acute aortic syndrome ranges from approximately 3.5 to 6.0 per 100,000 people per year worldwide but more than 27 per 100,000 in older adults [1,2,3]. The most common acute aortic syndrome is acute AD, accounting for approximately 85–95% of all reported cases [4,5,6].

AD arises from a primary tear in the aortic intima, which can frequently be found in vascular segments that are exposed to abnormal stress or shear stress. Abnormal changes in the physiological pulsating flow environment are one of important factors that induce primary tearing. Elongation of the ascending aorta can give rise to a region that is affected by low and oscillating wall shear stress (WSS) [7,8]. Such abnormal changes in the flow environment can lead to a state of atherogenic phenotype for the endothelial layer [9,10,11]. Histological studies have also shown that the fibrin, laminin, and fibronectin in the arterial wall face dissolution under the long-term increase the WSS in the progress of adaptive dilation remodeling of the vascular diameter, which may cause the interlayer adhesion damage and elastic fiber degeneration [12,13,14,15]. At the same time, hypertension is associated with the hypertrophy of the arterial wall [16], leading to increased wall stiffness [17]. In addition, hypertension has a long-term effect on the Inner and one-third depth of the external media [18], where AD primarily occurs [19].

It is seen from the previous research that vascular wall damage can be characterized by penetrating wounds of the inner aortic wall and delamination between aortic layers. Several experimental studies have shown that much higher pressure is needed to induce dissection on healthy aortic tissue, of which the triggering pressure of dissection can reach as high as 600 mmHg. In contrast, even much lower blood pressure can cause the continuing development of tears if an inner aortic wound exists [20,21,22]. It can be seen that the trigger pressure of the dissection significantly depends on different types of vascular wall damage.

In vivo experiments with large animals have great research significance for investigating AD formation mechanisms. Tang et al. and Wang et al. achieved a 75% success rate for inducing type B dissection in canine samples by injecting adrenaline and making an initial tear on the arterial wall [23,24]. In the experiment of Guo et al., the initial tear feature was a transverse circumferential notch that covered one-third of the proximal descending aorta [25]. The delamination between aortic layers, termed the “entry pocket”, was also incised on the aortic circumferential direction to induce dissection development. However, due to the complexity of animal experiments and the difficulty of control, it is challenging to observe the specific development process.

In vitro studies that use compliance-matched idealized aortic phantoms may be more controllable to implement and cause less animal suffering. Coupling the in vitro and zero-dimensional model, Rudenick et al. and Soudah et al. analyzed the influence of tear size and hemodynamic changes on the pressure and flow rate in the true lumen (TL) and false lumen (FL) [26,27]. Birjiniuk et al. characterized the relationship between pressure and flow rate in the false lumen and carried out suggestions for the stratification of deterioration risk in patients with type B AD [28,29]. Brunet et al. presented a novel experimental method to observe the propagation of the arterial dissection using a porcine carotid artery, where a longitudinal notch was created from the inside. It was concluded from their experiment that high shear stress in the crack tip is a possible trigger for the propagation of the dissection [30]. However, another opinion is that the alternating stress of stretching and contracting acting on the crack tip is far more crucial than the shear stress [31,32].

Despite many advances in the comprehension of the underlying mechanisms of aortic dissection in the last two decades, there is still a lack of work concerning the mechanism of tear propagation due to its complexity and nonlinearity. The advantage of the in vitro dissection model is that the deformation of the primary tear and the early stage of dissection propagation can be precisely investigated. Nevertheless, to date, fabrication of a developable dissection aortic phantom has not been realized because of the complexity of making primary tears in the inner layer [28,33,34]. Our previously proposed brush-spin-coating method can be further developed for making a vascular model with an initial inner layer tear since the coating method can mimic the laminated structure and, therefore, can be used for fabricating the aortic phantoms with dissection features [35].

This work aimed to develop a method for fabricating silicone models with dissection features inside. In vitro experimental research was also carried out to analyze the delamination behaviors. The overall structure of the paper is as follows. Section 2.1 discusses the characteristic of AD in human aortic tissue. Section 2.2 describes the processing method of the silicone phantom model with an initial tear in detail. In Section 2.3 and Section 2.4, the experimental setup and the observing method are introduced step by step. The results of the tube pressure measurement computed tomographic (CT) scanning and laser imaging measurement throughout the development of AD are presented in Section 3.1 and Section 3.2. In Section 4, the possible key factors inducing the dissection are analyzed, and conclusions are drawn in Section 5.

## 2. Materials and Methods

### 2.1. Key Features of the Tear of AD in Human Aortic Tissue

**Figure 1:**
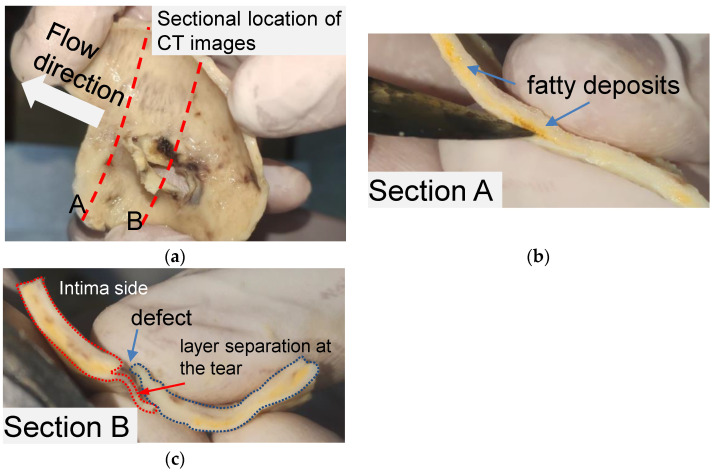
Anatomic structure of the aortic tissue and schematic diagram of the tissue at the breach. (**a**) A piece of formalin-soaked aortic tissue and its two cut positions. (**b**) Yellow fatty deposits showing an atherosclerotic layer on section A. (**c**) Vascular defect and layer separation around the tear on section B.

The structural features of the primary tear for the idealized phantom were modeled by observing a piece of human aortic tissue and the CT images of the same patient. The tissue was obtained from a 52-year-old man and soaked in formalin for 48 h. The patient gave written informed consent. Figure 1 shows the layout of aortic tissue from the intima side. The aortic tissue has been cut through the middle of the tear (section A) and 10 mm distal to the tear (section B) to observe the tissue morphology.

Pronounced delamination was observed in section A of the aortic tissue (Figure 1b). The yellow loose connective tissue between the media around the ostia of the rupture was identified as accumulated adipose tissue via pathological examination. As shown in Figure 1c, a defect was found on the intima side of the tissue, and a thin film of vascular tissue covered the defect. The thin vascular film extended only about 7 mm and then detached from the blood vessel, leaving a wound in the tissue. It should be noted that the aortic tissue was the dissection flap, which contained only the aortic intima and part of the media.

The aortic tissue observed in section B (Figure 1c) provides the basic anatomic structure for creating a primary tear on a silicone phantom. AD’s two critical anatomic features are the defect located on the intima layer and the separation begins from the defect between the layers. Accordingly, for a silicone phantom, the primary tear can be composed of a tear in the inner wall and an area of a tiny pocket between the silicone layers.

Based on the observation of human aortic tissue, four critical parameters were considered in the modeling to represent the dissection: the thickness of the TL and the FL, the area of the inner tear, and the area of the pocket-shaped layer separation. The manufacturing steps of tear-embedded silicone phantoms include the fabrication of an inner layer with a tear, the outer layer, and a tiny pocket between the inner and outer layers to form the primary tear region.

### 2.2. Fabrication Method of Tear-Embedded Silicone Phantom

**Figure 2:**
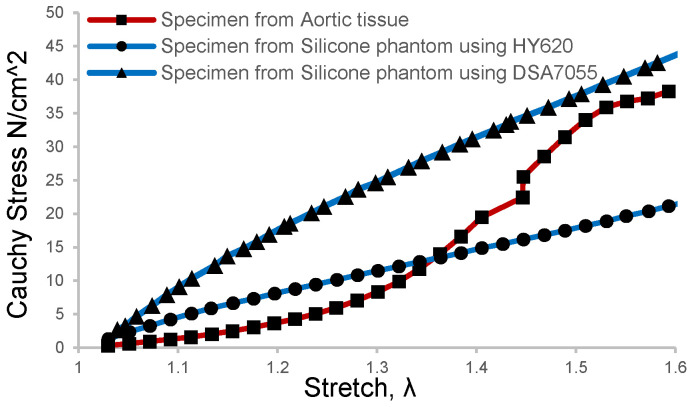
Tension test results of the aortic tissue sample and silicone phantom were processed with the brush-spin-coating method at the same size and position.

Brush-spin-coating was carried out to obtain a target thickness of 1.6 mm for the inner layer. In this study, two kinds of silicone materials were selected for the coating of the models, which were HY-E620 (Hong Ye E620, Shen Zhen Hong Ye Jie Technology Co., Ltd., Shenzhen, China) and DSA-7055 (DSA 7055, Guangdong Doneson New Materials, Guangdong, China). The mechanical properties of the two silica gels were measured and compared with the human aortic tissue obtained after surgery for aortic dissection. The aortic tissue of the patient was removed during the treatment of type A AD, then directly stored in a low-temperature environment of 4 °C in phosphate-buffered saline. Informed consent and ethical approval were obtained as well. In order to obtain more comparable results, patient-specific aortic arch silicone phantom models were made and then sliced into tension test strips at the corresponding positions of the obtained aortic tissue sample. The results of the direct tension test on the phantom samples and aortic tissue samples are shown in Figure 2. It can be seen that the tension test profile of DSA 7055 was closer to that of the aortic tissue when the strain rate was more significant than 1.5.

A recent ex vivo study on blood vessel expansion showed that pressure increments from 10 mmHg to 49 mmHg and 93 mmHg caused a circumferential strain rate of 1.32 and 1.50, respectively [30]. This means that the in vivo blood vessels underwent about 1.5 times the strain rate in a healthy state since the mean blood pressure of humans is 93.3 mmHg. As depicted in Figure 2, the elastic modulus of the silicone phantom DSA 7055 gradually approached the vascular tissue when the strain rate was about 1.5 rather than that of phantom E620. Meanwhile, the elastic modulus of our phantom was 0.72 MPa, which is slightly larger than that of normal tissue and approaching the value of elder or hypertensive patients [36,37]. This implies that the material can mimic the elder’s aortic tissue, which can be more easily developed into dissection. Thus the tear-embedded phantom model was made of silicone material DSA7055. The refractive index of DSA 7055 was 1.41, and its dynamic viscosity was approximately 3000 cps, which needed 90 min of curing and baking at 45 °C on a 3D-printed water-soluble cylinder model.

**Figure 3:**
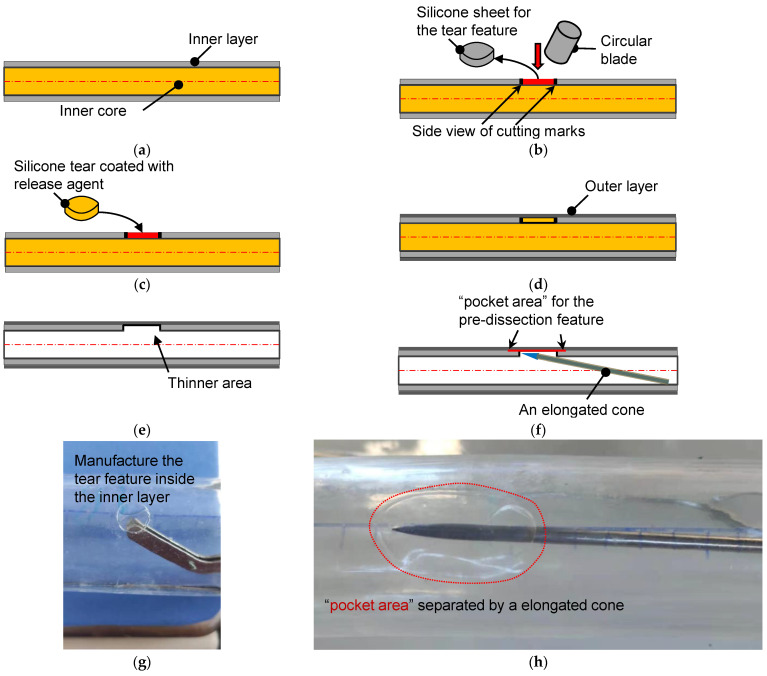
The schematic diagram for the fabrication of the aortic dissection phantom. (**a**) Side view of the inner core and its inner coating layer. (**b**) A circular blade cut a circular torn area. (**c**) The silicone sheet from the inner core was coated with a release agent and returned to the original position. (**d**) Coating of the outer layer on the inner layer with a targeted thickness. (**e**) Silicone tube with a torn area after the dissolution of the inner core. (**f**) Making pocket-shaped delamination between the inner and outer layers. (**g**) The actual operation is to remove the silicone sheet. (**h**) Creation of a pocket area between the layers by a cone probe.

The manufacturing process of a phantom with developable dissection features began when the inner layer’s coating was finished. Figure 3a shows the schematic diagram of the water-soluble inner core and the coated inner layer. A small circular piece of the inner layer was removed from the inner core, as depicted in Figure 3b. In cutting off the silicone sheet, a circular blade is important to avoid additional injury to the inner layer. From the side view, an empty region on the inner core’s surface was left after this operation. The circular empty area and the silicone tear were then coated with a release agent (Figure 3c), and the silicone tear was returned to its original height to perform the final coating of the outer layer (Figure 3d). After the silicone phantom was completely cured, the inner core was dissolved to leave the inner and outer silicone layers, which remained glued together at this stage. Subsequently, the circular area coated with the release agent could be easily removed from the inside. From Figure 3e, it is seen that the torn area was a thinner part that was composed of only the outer silicone layer. The silicone model’s thickness was similar to that of an aortic ulcer or porcine model of AD [25].

The pocket-shaped slight delamination can be created using an elongated probe with a conical head (Figure 3f). Figure 3g,h show the pictures for creating the initial and pocket-shape delamination in an idealized aortic phantom. Two silicone models with different sizes of pocket areas were produced, among which the small dissection (SD) was an elliptical area with major and minor axes of 16 mm and 12 mm, respectively. Meanwhile, the principal axes of the elliptical area of the large dissection (LD) were 20 mm, but the short axes were the same as that of the small ones. The high-transparency silicone material, named DSA-7055, was adopted for the fabrication. In order to capture the specific process of tear development in the axial direction, laser irradiation of the middle plane of the model and video filming of the entire process of tearing in the top view were used. Pre-experiments have shown that laser irradiation is prone to reflections and refraction, which affects the quantitative study of features near the specific tear. Therefore, an LD model was used for the laser experiment, and an SD model was used for the CT scanning experiment. In addition, five more silicone models of the same tear size but without the interlayer delamination feature of the primary tear were fabricated as the control group. Due to limitations in CT room booking, all control models in this study were performed in a laser experiment.

### 2.3. Experimental Setup of the Laser-Enhanced Observation

**Figure 4:**
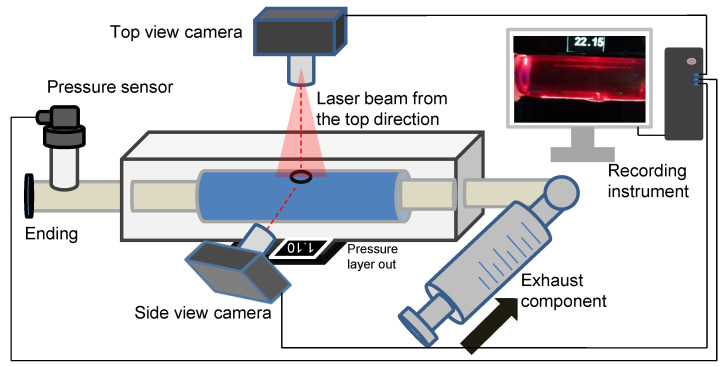
Schematic diagram of an experimental platform for laser-enhanced observation.

The in vitro experiment to investigate dissection in the idealized aortic phantom was conducted in a steady state. Figure 4 shows the essential elements of the in vitro experimental circulation. The experimental device contained three main parts: a pressure sensor and recording instrument, a laser-enhanced observation system, and a pipeline circulation with a dead-end for pressurization (Figure 4). The injected working medium was water at 20 degrees Celsius. Air exhaust components were used to eliminate all bubbles for the initial experimental condition. A pressure transmitter, which converted the pressure data into a 4–20 mA current signal, was recorded in real-time at a refresh rate of 0.1 s. Alterations in the morphology of the idealized aortic phantom and in the pressure were documented with cameras to ensure time-synchronized output.

The specific steps of laser-enhanced observation are as follows. At the beginning of the experiment, all bubbles were eliminated. Then, the pressure was adjusted to 2.5 kPa by twisting the screw. Subsequently, the recording was turned on, and the screw was manually rotated. The pressure readings in the tube lumen gradually increased during the twisting process, which was also recorded in the camera footage. The pressurization process then stopped when the experimenter observed the development of an interlayer or a rupture at the interlayer. The recording process continued until the development of the tube wall interlayer was stable again, or a rupture had occurred.

### 2.4. Experimental Setup of the CT Imaging

**Figure 5:**
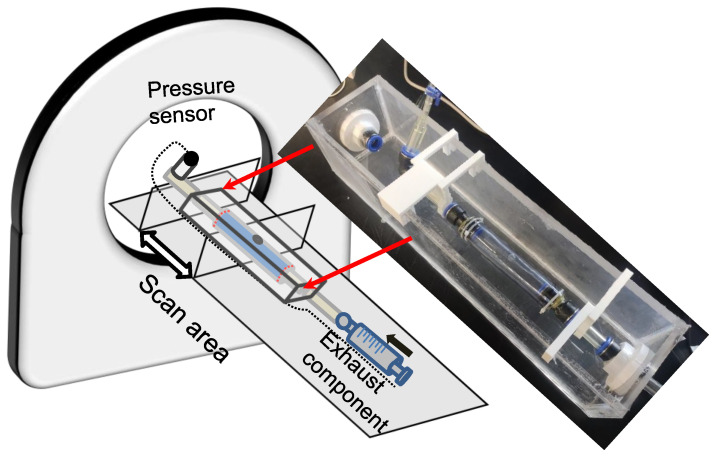
Schematic diagram of the experiment with CT scanning to capture the development of dissection.

CT scanning was conducted to investigate the volume and wall thickness alteration during the development of wall dissection. The experimental pipeline was settled on the CT scanning equipment as depicted in Figure 5 (X-ray equipment for CT; SOMATOM Force, Siemens Healthcare GmbH, Erlangen, Germany). The scope for scanning was targeted at the silicone phantom. The direction of the scan coincided with the phantom’s axial direction, and 15 mL iodixanol of contrast agent was added to 1000 mL of water and then filled into the pipeline. The in-plane accuracy of the CT images was 0.1563 × 0.1563 mm, and the interval between the in-plane sections was 1 mm. The tear-embedded model for scanning was an SD with a smaller pocket area around the internal tear.

In pressurization, the measurements could only be obtained before flow injection and CT scanning to avoid injury to the operations. Experimental pressurization and CT data extraction were performed alternately. The experimenter entered the CT control room to increase the pressure and quickly left the room while another operator immediately started the CT scan. If the CT scan revealed wall damage developing into a dissecting structure, then a continuous CT scan was maintained until the termination of the dissection process.

## 3. Results

### 3.1. Deformation of the FL and TL during the Development of Dissection

Pressurization experiments were performed for normal silicone phantoms and phantoms with initial damages. When the normal silicone tube was pressurized over 30 kPa, all the models were ruptured. In contrast, pressurization delaminated the tube models containing the feature of the primary tear into dissecting models.

**Figure 6:**
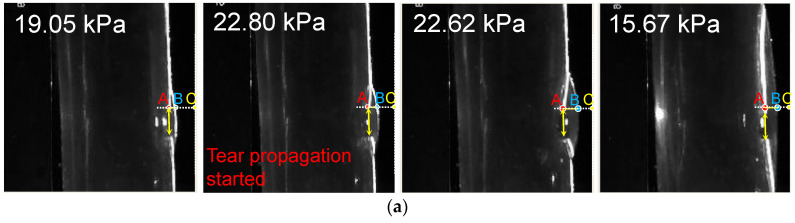

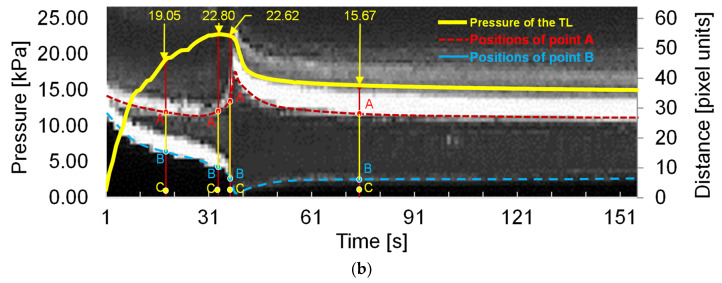
Main results of the laser-enhanced observation experiment. (**a**) Positions of the inner and outer layer during the pressurization. (**b**) Pressure recording and sequential images of the flap and outer layer positions during the pressurization. The left axis of Figure 6b is the pressure of the experiment, and the right side is the height scale of the picture, which is in pixels. The four experimental moments in Figure 6a are marked in Figure 6b using yellow arrows. In temporal latitude, point A constitutes the change in position of the inner layer of the membrane during the experiment, indicated by the red dashed line. The blue dashed line indicates the outer layer of the model.

The laser-enhanced observation was used to track the interfaces of TL and FL during the dissection development. Figure 6a shows four side views at different pressures. The first pressure state of 19.05 kPa was a moment in the gradual pressurization process. Pressure state 22.80 kPa was the pressure when the dissection started to develop. It is possible to qualitatively distinguish the increase of the tear at the 22.80 kPa from that of the pressure of 19.05 kPa.

There was no significant change in the line pressure from 22.80 kPa to 22.62 kPa within 3 s of the dissection starting to develop. However, the process underwent a dramatic increase in the volume of the FL. When the FL development process of the dissection was finished, the pressure of the line was stabilized at 15.67 kPa.

In order to observe the deformation of FL and TL during the pressurization, the temporal variation of tube pressure and deformation for the outer and inner layers are plotted in Figure 6b. The temporal deformations of the outer and inner layer are expressed using the variations of distance BC and AC indexed in Figure 6a, where A refers to the point of the edge of the internal tear, B is the intersection point of the transverse dashed line and the outer interface, and C is 3 px away from the largest-deformation point of B.

It can be seen that the distance of AC gradually declined before the pressure reached 22.80 kPa. As the pressure reached 22.80 kPa, the distance of AC increased severely while the pressure remained at almost the same value. After 3 s, the distance of AC decreased fast, accompanied by a rapid drop of pressure. When the pressure dropped to 15.67 kPa, at the time of 81 s, the variations of AC became stable. The variation of AC indicates that there was a dramatic inward movement of the flap to TL during the development of FL.

In contrast, the distance of BC decreased rapidly before the pressure reached 22.62 kPa, suggesting that the FL volume increased quickly with the pressurization. After the FL reached its largest size, the distance of BC increased slightly and reached a stable state gradually, suggesting that the development of FL had finished.

### 3.2. Volume and Wall Thickness Variations during the Progress of Dissection

**Figure 7:**
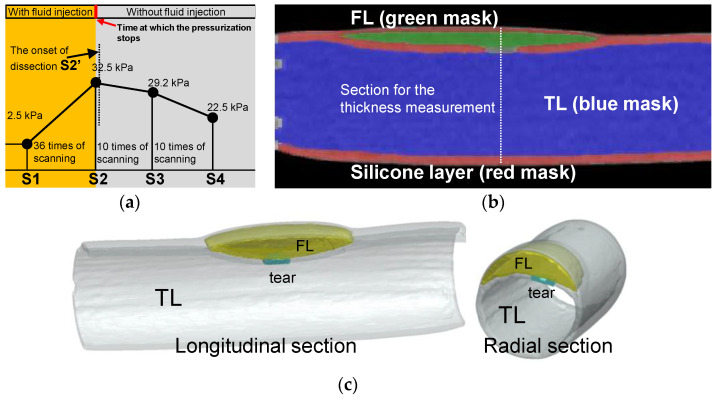
Experimental process of CT scanning and image reconstruction. (**a**) Pressure setting and the protocol of the CT scanning. (**b**) Reconstructed model of the longitudinal section from CT images at time point S2. (**c**) 3D reconstructed model of the longitudinal and cross-sectional view at time point S2′.

Pressurization and CT scanning were performed alternately until the experimenters discovered that the tear development process had begun. Figure 7a shows the time points for the data collection and timing for CT scanning operations. At the beginning of the experiment, 2.5 kPa was the pressure, so a set of CT scans were performed. The pressurization and scanning were alternated until the pressurization reached 32.5 kPa (36 times of scanning as depicted in Figure 7a), when the experimenter discovered that interlayer delamination had developed (the first sacan of 10 times of scanning as depicted in Figure 7a). After the delamination began to develop, the experiment entered the observation phase, and only CT scans were performed without further pressurization.

For data processing, this study positioned the initial pressure as S1 and the last CT scan before the onset of the dissection as S2. The CT that detected the development of the delamination was designated as S2′, one of the subsequent CTs during the development of the dissection was positioned as S3, and the final development status was positioned as S4. CT scan experiments were carried out under steady-state stepwise pressurization condition, which was the same as that in the laser tracking. Using the ScanIP module in the Simpleware software package (Simpleware Ltd., Exeter, UK), the volume of the TL and FL and the wall thicknesses of the silicone phantom were measured. Figure 7b gives a sectional reconstruction model at time point S3. Similar reconstruction models were obtained for all CT images at every scanning time point from S1 to S4. Figure 7c shows the reconstruction model at time point S2′ as dissection started to develop.

Regarding the error estimation of the experiment, the CT scanning was carried out three times at each scanning moment. However, FL was rapidly formed and increased at time point S2′. The scanning at time point S2′ was taken only once. For time points S1, S2, S3, and S4 (Figure 7a), three sets of CT data at each scanning point were selected for model reconstruction. On the other hand, the CT images at the moment S2′ were processed and constructed three times to avoid user errors.

**Figure 8:**
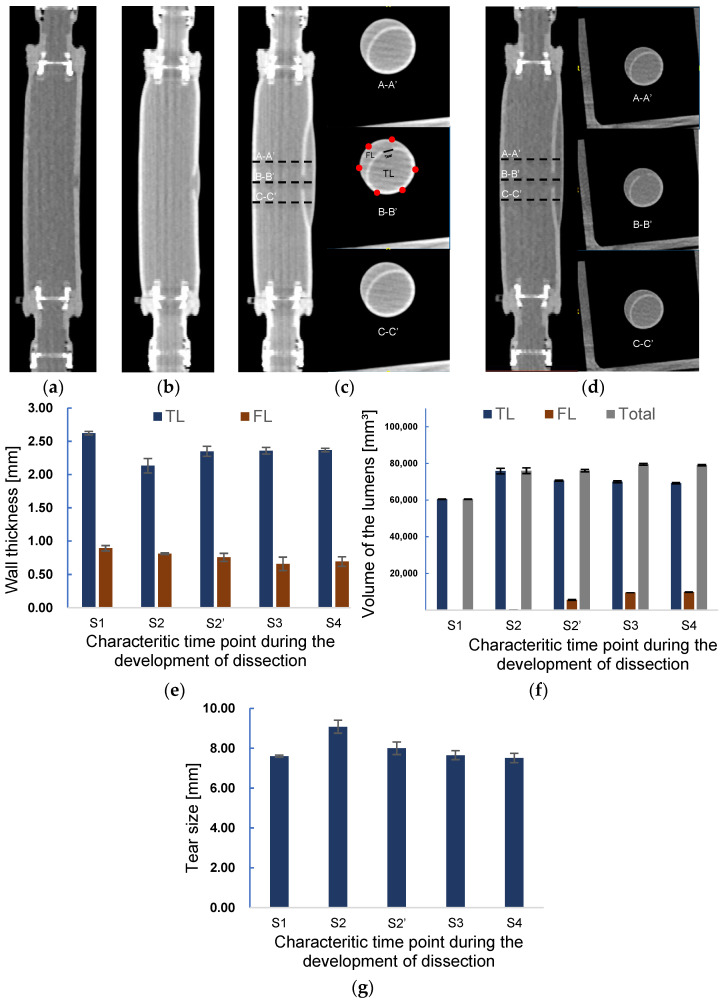
Axial CT images of at scanning time points (**a**) S1 and (**b**) S2. Axial CT images and the images of the cross sections A-A’, B-B’, and C-C’ at a scanning time point (**c**) S3 and (**d**). The distances between A-A’, B-B’, and C-C’ are 10 mm. Changes in the (**e**) wall thickness and (**f**) volume of TL, FL, and in total during the development onset of dissection. (**g**) Alteration of tear length during the development of dissection.

The sequential data of CT imaging were used to measure the thickness, and the three-dimensional model was used for the volume estimation. Figure 8 presents the images of CT scanning in the longitudinal direction at different time points in Figure 7a. It is observed from Figure 8a–d that the initiation and propagation of AD were the same as the observations in the laser lightning experiment. A small tear was observed in the inner layer from Figure 8a at the beginning of pressurization corresponding to the time point S1. At time point S2, a slightly enlarged FL can be observed from Figure 8b. The lumen’s radial views for three different positions, A-A’, B-B’, and C-C’, at the scanning moments of S3 and S4 are depicted in Figure 8c,d. The distance between the sections was set to 10 mm. The thickness of TL and FL, the volume of TL and FL, and the length of tear size were measured according to the cross-sectional images of the B-B’ position (B-B’ in Figure 8c).

Figure 8e shows the measured wall thickness at each scanning point. At the initial scanning time S1, the normal and tear wall thickness was 2.6 and 0.9 mm, respectively. In contrast, the average wall thickness of TL and FL at the moment S2 was 18.7% and 9.1% thinner than those of S1, respectively. Correspondingly, the tear size was enlarged by 19.5% in the process of increasing TL.

At time point S2′, as shown in Figure 8f, the volume of FL increased considerably, being 25-times larger than that at S2 due to the sudden delamination of layers. Meanwhile, the volume of TL was reduced by about 6.9%. The volume of the phantom at time point S2′ still remained the same as that at S2. The diameter of the tear decreased by 11.9%, approaching the original size. The wall thickness of TL recovered to 92% of the original one after decreasing at time point S2.

In addition, the dissection further spread along the axial direction after the S2 time point, resulting in a 78.6% increase in FL volume and a small decrease of TL at the S3 time point. Correspondingly, the wall thickness of FL decreased, but TL wall thickness remained almost the same as that at the previous time point S2′. The expansion of FL stopped at the time S4. Except the slight augment of FL, the TL volume, wall thickness of the TL and FL, and tear size remained nearly the same as those at the previous time stage S3, indicating that the propagation of dissection had ended.

## 4. Discussion

### 4.1. Experimental Biomechanical Study for Aortic Dissection

Many studies have been conducted to quantify the strength of the aorta by means of stretching, bulge inflation, the peeling test, the trouser test, the direct tension test, and the in-plane shear test [38]. Iliopoulos et al. and Manopoulos et al. found that the circumferential tensile failure stresses in the anterior, posterior, right, and left lateral were not significantly different between the control, aneurysm, and dissection groups [39,40,41]. However, in the axial direction, there were significant differences in tensile failure stresses between the various types of samples, with the right lateral being higher than the anterior and the posterior side in control samples. At the same time, the left and right lateral were higher than the anterior side in the aneurysm samples, and the right lateral was higher than the left in the dissected samples.

Although uniaxial tensile tests provide valuable insight into the strength properties of aortic tissue, unidirectional tensile tests are limiting in representing in vivo loading conditions. Therefore, some studies have attempted to measure the mechanical properties of the aorta by means of bulge inflation tests. Sugita et al. reported the longitudinal weakest of the normal aorta under the bulge inflation test [42], which is close to the tensile test data, but the crack formation and crack development aspects of aneurysmal tissue still need further study [43]. Through bulge inflation experiments, Pearson et al. found that the fracture elongation of ascending aortic samples was significantly greater compared with isthmus samples [44].

Brunet et al. carried out a series of studies that were closer to physiological realism than the bulge inflation experiments. Brunet et al. designed a custom tension inflation device that fit into an X-ray microscopic imaging setup. The X-ray tomography device allowed observation of the wall structure and incision behavior during carotid artery inflation. In the following pressurization experiment of a vascular lumen with an intima notch [30], when the pressure was continuously inflated to 687 mmHg (91.6 kPa), widening and deepening of the notch were observed. However, the vascular still had difficulty developing a dissection structure even though the experimental pressure was increased to approximately 687 mmHg. This may be related to the form of the intima notch [30].

A reasonable feature of the tear may be one of the critical factors in whether the tearing process can be triggered under experimental conditions. During the formation phase of aortic dissection, a long-term abnormal hemodynamic environment may lead to wall damage developing gradually. As a result, by the time the dissection is triggered, the mechanical properties of the vessel wall may already be damaged, and then pressure incensement is often considered an essential trigger for the onset of dissection. Taking into account how the in vivo experiments of dissection were produced in the previous study by Guo et al. [25] and the dissected tissue samples obtained in this study, the key features of the tear were designed. At the same time, the previous study proposed the corresponding method of producing the wall injury in the form of tearing and pocket interlaminar separation. In the following experimental research, the tear model developed into a sandwich structure during pressurization, suggesting that further exploration of injury forms may help to improve the physiological realism of dissection-triggered experiments.

### 4.2. Analysis of the Forces during the Development of Dissection

The mechanism of the interlayer forces during tear development has been described by previous studies [31,32], but there is a lack of experimental evidence. The present study, although differing in material properties from aortic tissue, may provide an analytical basis for the overall mechanical behavior of dissection development.

**Figure 9:**
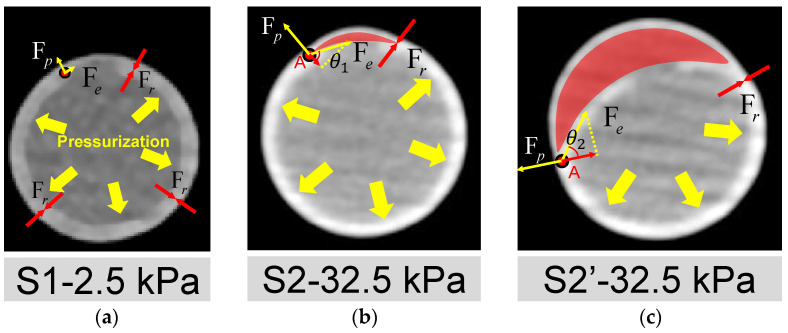
Analysis of mechanisms for the initiation and development of AD. Deformation of the cross-section of the silicone phantom and the forces acted on the wall (**a**) at the time point S1; (**b**) at the time point S2; (**c**) at the time point S2′. Fp, Fr and Fe are the pressure of the tube, the adhesion force of the silicone layers and the elastic tension of the inner silicone layer, respectively. The FL area is indicated by the red shading. A is the point where TL and FL are still stuck together. Fe and Fp act on point A, indicated by the yellow arrow. The projection of Fe in the opposite direction of Fp is the force that may cause interlayer detachment to start developing at point A.

The open area around the intima tear is directly associated with whether the dissection can be triggered. The mechanisms may be explained by the force balance depicted in Figure 9, where Fp, Fr, and Fe are the tube pressure, adhesion force of the interlayer, and elastic tension force of the silicone layers, respectively. At the early stage of pressurization (Figure 9a), the silicone wall expanded with pressure. When the experimental pressure was inflated to 32.5 kPa, as shown in Figure 9b, a small dome area was formed (red region in Figure 9b) above the internal tear due to a thinner pipe wall at the outer side of FL. More fluid inside the lumen may flow into the small dome, breaking the force balance on the flap’s two sides and the edge point A.

The force of the inward pulling effect at point A is the component of the Fe opposite to Fp, as shown in Figure 9b,c. When the pressure increased, it led the combined force of Fe and Fp to be more significant than the resistance to tearing Fr at Point A. Then, the dissection began to develop, corresponding to the period of S2 to S2′. As can be seen in Figure 8f and Figure 9c, during this period, the dissection was mainly in the circumferential direction, which confirms the viewpoint of Mikich and Qiao et al. [31,32].

The influence of the size of the pocket area between layers can be explained by the analysis of force balance acting on the edge point A as well. The increase in pocket area made the flap flatten. At this condition, the angle θ at point A will increase, as depicted in Figure 9c, which means a lower pressure increment can trigger the dissection.

The tension force Fe is related to the thickness of the flap and maybe even the shape of the local vascular morphology. If the flap thickness increases, then the Fe will increase. As previous studies have reported, a thicker flap may lead to a more severe dissection level [22,25]. In addition, the properties of the aortic flap in chronic AD may also need to be further considered because the stiffening of the dissected flap is common in AD patients [45]. In addition, from Figure 8c,d, it is seen that there was no significant difference for the edge of FL in the circumferential direction at S2′ and S3, both of which ended at the half circle of the cross-section. However, the FL developed in an axial direction considerably at S3 time point, implying that the development of AD may first occur in the circumferential direction and end while reaching a new force balance [46]. The feature of developing first in the circumferential direction is most likely related to the radius of curvature of the vessel wall. This should interest investigators because some common areas of proximal tear, such as the lateral aspect of the descending aorta, the aortic arch, and the proximal area of the brachiocephalic artery, have a far more complex radius of curvature feature than the straight tube features in this experiment.

The present study also suggests that the “straining” pattern of the intimal wall may be a promising screening indicator for aortic dissection risk. It is likely that minor vascular injuries or even small tears in the intimal wall of the aorta exist prior to the onset of dissection, but such tears are difficult to detect with conventional imaging techniques [47,48]. In this study, the strained state of the wall (affected by tension force Fe) can assist in clinical screening for the early stage of AD. For example, if the CT angiographic findings of an intermural hematoma occurred, the aortic vessel also showed morphological features of a wall being straightened, as shown in Figure 9 of this study. At the same time, its outer wall also experienced more significant distension than the adjacent location. Therefore, based on the present experimental findings, the risk of dissection needs to be taken seriously.

**Figure 10:**
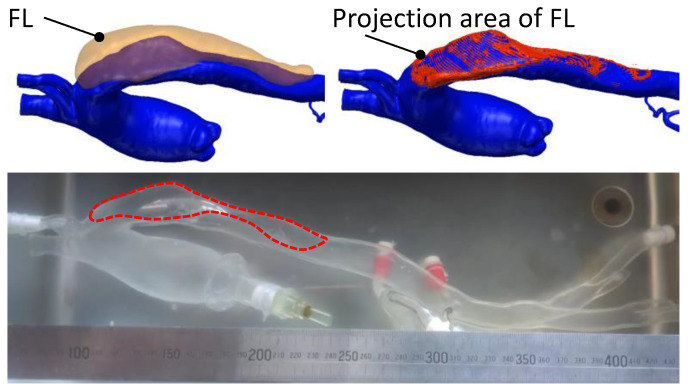
Personalized aortic silicone phantom with interlaminar damage structures.

This dissected phantom has good clinical translational benefit prospects. Traditional dissected models usually make the FL into a channel. The present study allows for structural changes in the dissection from injury to the dissected lumen and, therefore, more closely resembles the actual process of AD. It is also possible to quickly test for blood flow and pressure changes within the interstitial layer. Therefore, this model’s most direct clinical translation is to be used by interventionalists as a teaching tool for clamping stent placement. At this stage, the technique is available for personalized models, as it is depicted in Figure 10. Clinicians can perform a series of studies on this model regarding stent anchorage zones and techniques involving in vivo and ex vivo stent fenestration.

### 4.3. Limitations

Despite the detailed observation of the dissection propagation process in the study, several shortcomings should be investigated further. The material’s elastic modulus did not show the nonlinearity of actual aortic tissue. A phantom with a nonlinear elastic modulus or anisotropic mechanical properties may be fabricated by coating silica gel fibers on the model’s surface. Another noticed limitation of this study is the dissection-triggering condition. In order to facilitate the observation of results, stepwise steady-state pressurization boundary conditions have been employed in most studies on the interlayer triggering of AD [20,30]. In future work, AD development in various pulsatile pressure conditions should be carried out to see what kind of pulsatile pattern is the most dangerous for development.

For simplicity, we employed water as the injected fluid in the two observation experiments. In a further study, glycerol and water should be used to obtain a fluid with the same viscosity as the blood. At the same time, the current research ignores the effect of personalized aortic structure on a primary tear, of which tortuosity, curvature, or tapering of the aorta may influence the sizes and positions of the primary tear [8,49]. By coupling personalized aortic structure fabrication techniques with this study [35], more realistic AD structures can be achieved, where thoracic endovascular repair (TEVAR) may be simulated.

In terms of subsequent development of the method, the intermural hematoma model is also one of the directions. Hypertension and aortic intermural hematoma are not uncommon in the advanced age group. In this model, the intermural hematoma aortic phantom model can be obtained by reducing the tear characteristics or using highly permeable silicone instead of tear fabrication. Future studies of tear triggering and hematoma volume on wall morphology using the intermural hematoma phantom model, and even the assessment of high-risk hematoma location, will be carried out gradually based on the technical solutions of this study.

## 5. Conclusions

We fabricated an in vitro silicone model with primary tear and adhesion damage, which developed a dissection via a stepwise pressurization experiment. The experimental approach allows for clearly tracking the initiation and propagation process of dissection.

At the initiation stage, only the volume of TL increased with the pressurization. There were no significant size changes for the primary interlayer damage. At the propagation stage, a severe increase of FL volume was observed, accompanied by rapid inward movement of the flap and the shrinking of the tear, leading to the decline of pressure in the silicone phantom. Meanwhile, the thickness of the FL gradually decreased as the dissection developed, which increased the risk of FL rupture. The size of the primary pocket-shaped tear significantly influenced the pressure required to trigger dissection. The larger the primary pocket-shaped area, the lower the pressure required.

The experimental results indicate that the primary tear and the weakening adhesion of the vessel layers are key factors in AD development. These two features are also frequently observed in patients with the acute aortic syndrome. This study suggests that some forms of primary damage to the arterial wall, such as aortic ulceration with aortic hematoma, which weakens the interlayer adhesion of the aorta, should be considered as early risk predictors of AD. This type of dissected model will have good prospects for clinical translation in areas such as interventional surgery rehearsal.

## 6. Patents

Qing-Zhuo Chi, Li-Zhong Mu, Ying He, and Zhen Cao. Manufacturing method of personalized extracorporeal interlayer physical model. China Invention Patent, CN112669687A.4[P], 16 April 2021 (Licensed).

## Data Availability

Not applicable.
